# Innovations in Designing Hydrogels for Advanced Wound Dressing Applications: An Editorial Review

**DOI:** 10.3390/gels11050332

**Published:** 2025-04-29

**Authors:** Kannan Badri Narayanan, Rakesh Bhaskar

**Affiliations:** School of Chemical Engineering, Yeungnam University, 280 Daehak-Ro, Gyeongsan 38541, Gyeongbuk, Republic of Korea

## 1. Introduction

Hydrogels are highly versatile biomaterials that play a crucial role in personal wound care and regenerative medicine. Polymeric networks, which are capable of holding significant amounts of water, serve as supportive scaffolds for mammalian cell growth and as carriers for bioactive molecules, including antimicrobial agents and therapeutic drugs [1]. The design of hydrogels for wound care applications, including the treatment of chronic wounds and burns, necessitates several fundamental properties, such as a high water content, the ability to maintain a moist wound environment, gas permeability, and biocompatibility [2]. The physicochemical and biological properties of hydrogels are primarily determined by their polymer composition (natural, synthetic, and/or hybrid), choice of solvent, crosslinking strategy, porosity, pore size, swelling capacity, biodegradability, surface functionalization, and topographical features. Additionally, hydrogels designed for wound dressings should possess mechanical flexibility, adhesiveness, and controlled drug-release capabilities to ensure that they achieve optimal therapeutic efficacy [3]. In polymeric crosslinking, avoiding toxic chemical crosslinkers is crucial to ensuring biocompatibility while enabling the achievement of tunable mechanical and biological properties. Recent advancements in hydrogel technology have leveraged sophisticated fabrication techniques, such as ionizing radiation and electron beam irradiation-mediated hydrogel crosslinking, which offer advantages such as biocompatibility, sterilization, and precise control over the hydrogel’s properties. Additionally, the incorporation of nanomaterials, including nanoparticles and nanofibers, has significantly enhanced the mechanical robustness and therapeutic functionality of hydrogels. These advanced hydrogels, with engineered nanotopographical features, play a pivotal role in influencing the wound-healing process by modulating cellular responses, such as adhesion, proliferation, migration, extracellular matrix (ECM) deposition, and angiogenesis [4,5]. Moreover, the micro- and nanoscale patterning of hydrogels has demonstrated significant potential in inhibiting bacterial adhesion, proliferation, and biofilm formation, thereby reducing the risk of infections and promoting efficient wound healing [6,7]. Thus, understanding these intricate hydrogel design principles and functionalities is crucial for developing next-generation wound dressing hydrogels that enhance the wound healing efficacy and improve patient outcomes. This Special Issue focuses on synthetic and natural hydrogels, both with and without the incorporation of nano- and microstructured materials, for use in various biomedical applications and clinical practice.

## 2. Overview of the Papers Published in This Issue

This Special Issue, titled “Designing Gels for Wound Dressing”, features four research articles and two review articles that discuss the advancements in biomaterials for wound care and tissue engineering. These contributions explore innovative fabrication techniques, material characterizations, and their potential for clinical applications.

### 2.1. Key Highlights from Research Articles

Narayanan and colleagues fabricated natural scaffolds for improved skin tissue engineering applications. Their study investigated the utilization of bacterial cellulose as a natural polysaccharide hydrogel, which was functionalized with polydopamine to enhance its adhesiveness and biocompatibility [8]. The functionalization of the bacterial cellulose with polydopamine nano-/microspheres altered the scaffold’s surface topography and improved the adhesion and proliferation of NIH/3T3 murine fibroblasts. In another study, Zhang and colleagues developed a PEG-based hydrogel integrated with zinc oxide nanoparticles (ZnO NPs) to achieve enhanced hemostatic and procoagulant efficacy in treating severe soft tissue bleeding. The dual-network structure of the hydrogel, formed through amide bond formation and zinc ion chelation, enhanced its mechanical strength and adhesion properties. The controlled release of zinc (Zn^2+^) ions further promoted coagulation and hemostasis, making the hydrogel highly effective for acute bleeding treatments in both visceral and peripheral injuries [9].

Chaikhunsaeng et al. [10] fabricated biocomposite hydrogel films for biomedical applications from bacterial cellulose (BC) and thin-shell silk cocoon (SC) through a simple, cost-effective, and sustainable method. In the production of the S/BC films using *Acetobacter xylinum*, SC was incorporated into the culture medium containing either mature coconut water (CW) or coconut skim milk (CM), resulting in the formation of BCW and BCM films, respectively. The tensile strength and elongation at the break of the BCM films were significantly higher compared to the BCW films. Moreover, the incorporation of SC enhanced the adhesion, proliferation, and cell-to-cell interactions of the Vero (kidney epithelial cells of the African green monkey) and HaCaT (human skin keratinocytes) cells, making the biocomposites highly suitable for wound dressing and tissue engineering applications [10]. Collagen-based biomaterials are widely used in tissue engineering and cosmetics, yet clinical evidence on the safety of injectable collagen products remains limited. To address this issue, Koo and colleagues developed a novel sterilization technique involving double filtration (300 kDa and 100 kDa cut-off sizes) and low-temperature steam sterilization (40 °C, 0.7 atm for 40 min) for a type I collagen gel. This method preserved the structural properties of the collagen gel, ensuring its sterility, biocompatibility, and protein integrity. Both in vitro and in vivo studies confirmed its efficacy as a safe and effective clinical skin booster [11].

### 2.2. Insights from Review Articles

In the review article published by Arabpour and colleagues, the authors reviewed the use of various hydrogel-based nanoparticle systems to overcome the anatomical barriers in ocular drug delivery, including the corneal epithelium, blinking reflex, aqueous–blood barrier, and retina–blood barrier. They also discussed the challenges facing drug absorption and highlighted the integration of nanotechnology with polymeric hydrogel systems, such as nanosuspensions, nanoemulsions, and pharmaceutical nanoparticles. These systems offer effective, non-invasive solutions for targeted site-specific drug delivery to treat ocular diseases [12]. Similarly, Chylinska and Maciejczyk [13] provided a comprehensive analysis of the role of hyaluronic acid (HA) in topical anti-aging formulations, nutricosmetics, HA-based fillers, and skin biostimulators. Furthermore, they explored various HA-based dressings, including hydrogels, sponges, membranes, and films, highlighting their therapeutic potential in wound healing applications. Their review emphasized the importance of hyaluronic acid-based hydrogels in promoting cell proliferation, ECM remodeling, and hydration, making them valuable biomaterials for regenerative medicine.

## 3. Common Research Limitations and Technological Challenges

Hydrogel-based wound dressing systems have emerged as a leading class of biomaterials in advanced wound care due to their unique properties, including their high water content, biocompatibility, and tunable physical and chemical properties. In addition to providing a moist environment which is favorable for wound healing, they serve as multifunctional platforms for drug delivery, antimicrobial protection, and tissue regeneration. Recent innovations have focused on the incorporation of bioactive molecules, stimuli-responsive polymers, controlled drug delivery systems, and nanomaterials into hydrogel dressings to enhance their therapeutic efficacy. Despite these promising advancements, several research limitations and technological challenges continue to hinder their widespread clinical translation and commercialization. These key challenges include the following:

1. Biomimicry and structural integrity: Designing hydrogels that accurately mimic the native extracellular matrix (ECM) of the skin’s architecture and dynamic physiological functions remains a significant challenge. Ensuring the appropriate combination of flexibility and mechanical strength is crucial for the effective treatment of different types of wounds.
2. Mechanical stability and durability: Many hydrogels exhibit insufficient mechanical strength and low stability, limiting their use in high-stress wound environments or for extended wear.
3. Biocompatibility and immunogenicity: Ensuring high biocompatibility while minimizing immunogenicity and antigenicity is essential to prevent adverse host responses.
4. Controlled and sustained drug delivery: Hydrogels must support the stable encapsulation and controlled release of diverse bioactive molecules (e.g., growth factors, antibiotics) at therapeutic concentrations. Delivering sub-therapeutic concentrations can contribute to the emergence of drug-resistant microbial strains.
5. Antimicrobial resistance (AMR): The improper release of antimicrobial agents from hydrogels can inadvertently promote the emergence of resistant microbial populations within the treated wounds.
6. Scalability and quality control: Maintaining consistency in physicochemical and biological properties during large-scale production remains a significant hurdle. Batch-to-batch variability can affect a product’s efficacy and regulatory approval.
7. Environmental and ethical considerations: The sourcing of raw materials, use of animal-derived components, and environmental impact of manufacturing processes are growing concerns that must be addressed.

Addressing these complex challenges requires interdisciplinary collaborations among experts in polymer science, materials engineering, biochemistry, microbiology, and biomedical sciences. To facilitate clinical translation, the fabrication of hydrogel wound dressings must prioritize sustainability, scalability, and cost-effectiveness, while simultaneously enhancing their mechanical and biological performance. Moreover, robust validation through preclinical models and well-designed human trials is essential to demonstrate the safety, efficacy, and regulatory compliance of such hydrogels.

## 4. Impact and Relevance of the Special Issue Contributions

This Special Issue provides significant insights into both the fundamental and applied aspects of hydrogel technology, emphasizing innovative fabrication techniques and functionalization strategies for transforming the field of wound healing and tissue regeneration. The collective findings are highly relevant to researchers, healthcare professionals, and product engineers working to translate laboratory innovations into effective clinical solutions.

The contribution of Narayanan et al. [8] highlights the synergistic use of natural biopolymers and bioinspired functional adhesive proteins such as polydopamine (PDA), paving the way for next-generation skin substitutes that closely mimic the architecture of the extracellular matrix (ECM). This approach offers a safer, more sustainable, and efficient alternative to conventional synthetic wound dressings. The work by Zhang et al. [9] introduces a PEG-based hydrogel embedded with ZnO NPs, enabling the controlled release of zinc ions to enhance clotting responses. This innovation addresses a key limitation of conventional hydrogels by providing effective hemostasis for both visceral and peripheral injuries, thereby broadening the clinical relevance of hydrogel-based materials. In another notable study, Chaikhunsaeng et al. [10] develop biocomposite hydrogel films using natural components such as bacterial cellulose with coconut water and skim milk. This economically viable and environmentally sustainable approach offers a promising model for low-income countries by integrating circular economy principles and leveraging agricultural and natural waste to produce high-value healthcare products. The study by Koo et al. [11] addresses a critical aspect of developing clinical biomaterials—sterilization. The researchers develop a novel sterilization method involving double filtration and low-temperature processing, which preserves the structural integrity and biological activity of injectable collagen gels. This advancement holds significant promise for the commercial-scale production of collagen-based injectable skin boosters, dermal fillers, and wound care gels. The review by Arabpour et al. [12] investigates the underexplored field of ocular drug delivery using hydrogel systems. It positions hydrogels as versatile carriers for non-invasive ophthalmic therapeutics, expanding their utility beyond skin-based applications and addressing the anatomical and physiological challenges of the eye. Similarly, the review by Chylinska and Maciejczyk [13] examines hyaluronic acid (HA)-based hydrogels in both cosmetic and wound healing applications. Their comprehensive analysis emphasizes the multifunctionality of engineered HA-based hydrogels as promising platforms for wound healing, drug delivery, and tissue regeneration.

Collectively, the contributions of this Special Issue underscore a variety of novel strategies and emerging approaches in the design of hydrogel-based wound dressing materials. The featured works emphasize multifunctionality, clinical translation, environmental sustainability, and accessibility—hallmarks of the next generation of biomaterials which are tailored for personalized and precision medicine.

## 5. Conclusions and Future Perspectives

In conclusion, the studies featured in this Special Issue demonstrate significant developments in the design and application of biomaterials, particularly those incorporating unique surface topographies and nano-/microstructured metal oxides, for use in wound dressing and tissue engineering. The innovative strategies presented in these articles highlight the potential of various natural and synthetic hydrogels in wound care and regenerative medicine, paving the way for advanced therapeutic interventions and improved patient outcomes. Looking toward future advancements, several promising research directions are proposed to enhance the clinical translation and therapeutic efficacy of hydrogel-based wound dressings:

1. The development of multifunctional hydrogels capable of the controlled and sequential release of multiple bioactive agents, such as antimicrobial, anti-inflammatory, and growth-promoting agents, to support the distinct phases of wound healing.
2. The incorporation of natural multi-drug-resistant antibacterial agents, including bacteriophages, into hydrogel matrices which are designed for targeted, stimuli-responsive release triggered by microbial virulence factors, thereby minimizing disruption to the beneficial native microbiota.
3. The design of biodegradable, self-healing hydrogels with minimal scar formation and enhanced mechanical durability, achieved through dynamic covalent or supramolecular interactions, enabling autonomous repair and prolonged in situ functionality.
4. The incorporation of oxygen-generating compounds, such as calcium peroxide or manganese dioxide, into hydrogels to improve their oxygenation in hypoxic wound environments, thereby promoting angiogenesis, collagen deposition, and overall tissue regeneration.
5. The engineering of anti-biofilm hydrogels incorporating quorum-sensing inhibitors and biofilm-disrupting agents to effectively combat chronic wound infections and facilitate accelerated healing.

Overall, the continued innovation in the design and development of biocompatible and hemocompatible hydrogels—through the exploration of diverse materials, compositions, crosslinking methods, and functionalization strategies—will provide valuable insights for researchers aiming to create next-generation wound dressings with multifunctional capabilities and diverse biomedical applications.
